# Patch Testing and Psychosocial Burden in Adults With Facial Dermatitis: A Cross-Sectional Observational Study

**DOI:** 10.7759/cureus.116473

**Published:** 2026-09-18

**Authors:** Garima Wadhwa, Sanjeev Gupta, Aneet Mahendra, Aditi Dabhra

**Affiliations:** 1 Dermatology, Maharishi Markandeshwar Institute of Medical Sciences and Research, MMDU Mullana, Ambala, IND

**Keywords:** contact allergen, contact allergic dermatitis, patch test, psychosocial impact, skindex

## Abstract

Background

Facial dermatitis is a common inflammatory dermatosis with multifactorial etiology and occurs due to repeated exposure to cosmetic, environmental, and occupational triggers. Because of its visible nature and chronic relapsing course, it may substantially impair psychosocial well-being and quality of life.

Objective

The primary objective was to study the clinical profile of patients with facial dermatitis and identify clinically relevant contact allergens. Secondary objectives were to assess psychosocial burden using the Skindex-16 questionnaire, evaluate its association with relevant clinical features, and explore associations between patch-test positivity and socio-demographic and clinical factors.

Methodology

This tertiary hospital-based cross-sectional observational study included 50 adults with facial dermatitis attending the dermatology outpatient department at Maharishi Markandeshwar Institute of Medical Sciences and Research (MMIMSR), Mullana, India. Detailed exposure history and clinical evaluation were followed by patch testing with the Contact and Occupational Dermatoses Forum of India (CODFI)-approved Indian Standard Battery, Cosmetic Series, and Fragrance Series. Reactions were interpreted according to International Contact Dermatitis Research Group criteria, and clinical relevance was assessed using the C=Current Relevance, O=Occupational, A=Accidental, D=Doubtful Relevance, E=Exposed, X=Unknown (COADEX) system. Psychosocial burden was evaluated using the Skindex-16 questionnaire.

Results

Of the 50 participants, 37 (74%) were female, and the most common age group was 31-40 years (40%). Disease duration of more than six months was present in 32 (64%) patients. Cheeks (84%), forehead (76%), and eyelids (68%) were the most frequently involved sites. Pruritus was reported by all patients. Sun exposure (92%), heat/sweating (56%), seasonal variation (54%), and hair-dye exposure (50%) were common aggravating factors. Positive patch-test reactions were observed in 12 (24%) patients, with 15 reactions involving nine distinct allergens. Paraphenylenediamine was the most frequent allergen (26.7%), followed by benzyl alcohol, benzyl salicylate, and bronopol (13.3% each). 8/9 distinct allergen types (88.9%) were classified as having cosmetic/topical relevance as per COADEX. Patch-test positivity was significantly associated with hyperpigmentation, hair-dye use, and history of atopy (p < 0.001, p = 0.002, and p = 0.023, respectively). The mean total Skindex-16 score was 39.4 ± 18.7, with the emotional domain showing the highest mean score (18.8 ± 8.9). Patch-test-positive patients had significantly higher Skindex-16 scores than patch-test-negative patients (median 69 vs. 34, p < 0.001). Patients with hyperpigmentation also had significantly higher total Skindex-16 scores (60.7 ± 14.7 vs. 28.4 ± 8.0, p < 0.001).

Conclusion

Patch testing is a useful diagnostic tool in facial dermatitis for identifying clinically relevant allergens, particularly those related to cosmetics and hair dye exposure. Facial dermatitis was also associated with substantial psychosocial burden, especially in the emotional domain. Comprehensive management should therefore include allergen identification, avoidance counselling, and psychosocial support. A comprehensive approach incorporating detailed clinical evaluation, exposure history, patch testing, allergen avoidance counselling, and psychosocial support may help optimize patient management.

## Introduction

Facial dermatitis represents a heterogeneous group of inflammatory dermatoses with diverse endogenous and exogenous causes [1-4]. Because facial skin is thin, highly exposed, and frequently in contact with cosmetics, topical preparations, airborne particles, and environmental irritants, it is particularly vulnerable to both sensitisation and irritation [2-4].

The reported prevalence and pattern of contact sensitisation vary considerably by study population, diagnostic criteria, and allergen series used. Indian estimates of facial dermatitis among dermatology outpatient department (OPD) attendees are limited, and studies specifically examining facial/cosmetic dermatitis demonstrate a substantial burden. For example, a North Indian study evaluated 400 patients clinically suspected of cosmetic dermatitis from a dermatitis-clinic population of 2,150 patients; 52.75% had a positive patch test to the suspected cosmetics [5]. A study by Rastogi et al. evaluating 50 patients with suspected cosmetic-induced facial dermatoses found paraphenylenediamine (PPD) to be the most frequent positive allergen (18%) [6]. Another Indian study by Yadav et al. of suspected facial contact dermatitis found 58 patients were patch tested, of whom 38 were patch-test positive; thiomersal, fragrance mix, and PPD were among the common allergens [7].

Although Jampuram et al. combined patch testing and quality-of-life assessment in patients with cosmetic dermatitis, their cohort included cosmetic dermatitis at multiple anatomical sites, and they assessed quality of life using the Dermatology Life Quality Index (DLQI) [8]. Although previous studies have assessed allergen profiles and quality-of-life in patients with contact or cosmetic dermatitis [8-11], evidence specifically integrating patch-test-defined allergens with Skindex-16-based psychosocial assessment in Indian patients with facial dermatitis remains limited. Thus, the present study addresses an important knowledge gap by characterising clinically relevant contact allergens in patients with facial dermatitis in India and also simultaneously assessing psychosocial burden using the Skindex-16 scoring.

In routine dermatological practice, recurrent facial dermatitis often presents with overlapping clinical morphology, making etiological diagnosis difficult on clinical grounds alone [2-4]. Patch testing is considered the gold standard investigation for identifying delayed type IV hypersensitivity reactions and has particular value in facial dermatitis, where allergens may be encountered through direct application, indirect transfer, airborne exposure, or occupational contact [4,6-8]. Identification of clinically relevant allergens helps guide counselling regarding avoiding allergens, reducing recurrence, and improving long-term disease control [5-8]. The burden of facial dermatitis extends beyond cutaneous symptoms. Because the face is central to self-image, interpersonal interaction, and social confidence, visible lesions may result in embarrassment, worry, low self-esteem, and impaired quality of life [8-11]. The present study was therefore undertaken to evaluate the clinical profile of facial dermatitis, assess the role of patch testing in identifying clinically relevant allergens, and measure psychosocial burden using the Skindex-16 instrument [12]. We have outlined the objectives of our study, as mentioned below:

Primary objective

The primary objective was to study the clinical profile of patients with facial dermatitis and to identify clinically relevant contact allergens.

Secondary objectives

The secondary objectives of this study included: 1. assessing the psychosocial burden associated with facial dermatitis using the Skindex-16 questionnaire, 2. evaluating the relationship between psychosocial burden and relevant clinical features, particularly hyperpigmentation, and 3. exploring associations between patch-test positivity and selected demographic, clinical, and exposure-related factors.

## Materials and methods

Study design and setting

This was a tertiary hospital-based observational cross-sectional study conducted in the Skin OPD of Maharishi Markandeshwar Institute of Medical Sciences and Research (MMIMSR), Mullana, India. The study was carried out over a period of 18 months from February 2025 to April 2026. Recruitment continued until 50 eligible patients with facial dermatitis were enrolled.

Sample size calculation

The sample size was calculated using the formula n = Z^2^pq/d^2^. Based on a patch-test positivity of 65.52% reported by Yadav et al. [7] in a comparable study, with a 95% confidence level and a relative allowable error of 20%, the estimated sample size was approximately 51 participants. Here, p = 0.6552, q = 1-p = 0.3448, and d = 0.20*p = 0.1310. Thus, n = 50.5, giving a minimum sample size of approximately 51 participants. So a target sample size of 50 participants was planned for the study.

Study participants

The inclusion and exclusion criteria have been listed below:

Inclusion Criteria

1. Adult patients ≥18 years, exhibiting signs of facial dermatitis; 2. Dermatitis over the face in quiescent stage (i.e., no active acute inflammatory eruption at the time of patch testing, with absence of significant erythema, edema, vesiculation, crusting, or oozing, and with clinically resolved facial lesions); 3. Regular users of topical personal care products and cosmetics over the face - this criterion was included because the study specifically aimed to identify clinically relevant contact sensitization in facial dermatitis, in which repeated exposure to cosmetic and personal-care products represents an important potential source of sensitization. Similar selection criteria have been used in previous studies evaluating cosmetic-induced facial dermatoses [6]; 4. Patients willing to provide written informed consent and attend follow-up sessions for patch testing.

Exclusion Criteria

1. Concomitant active skin disorders or signs of active inflammation like facial erythema, itching, photosensitivity, and any active lesions over the face like erythematous papules or plaques; 2. Patients on immunosuppressants; 3. Patients presenting with active skin infections like fungal, bacterial, and viral infections over the face; 4. Patients taking oral steroids (at a dose of prednisolone > 20mg/day); 5. Known hypersensitivity to patch testing materials; 6. Pregnant and lactating females; 7. Dermatitis in the acute stage (patch test will be carried out only after dermatitis has been controlled).

Facial dermatitis

Facial dermatitis was considered a clinical umbrella term for inflammatory dermatitis involving the face, characterised by erythema, scaling, pruritus, burning, or dryness. As there is no single standalone diagnostic criterion for facial dermatitis, diagnosis was based on clinical history, including detailed exposure history, and clinical correlation with the examination findings. Facial dermatitis broadly encompassed atopic dermatitis, seborrheic dermatitis, allergic contact dermatitis, irritant contact dermatitis, photoaggravated dermatitis, and airborne contact dermatitis, with specific diagnoses considered according to the respective established clinical criteria, mentioned as follows:

Atopic Dermatitis

Hanifin-Rajka criteria were used [13]. The major criteria include pruritus, typical morphology and distribution characterised by flexural dermatitis in adults and face/extensor involvement in infants and young children, a chronically relapsing course of dermatitis, and a personal or family history of atopy, including asthma, allergic rhinitis, or atopic dermatitis; at least three of these four major criteria are required. There are 23 minor criteria, of which at least three are required.

Seborrheic Dermatitis

Diagnosis was clinical, based on the characteristic clinical presentation of poorly defined erythematous patches or plaques with fine or greasy scaling, predominantly involving seborrheic areas such as the scalp, eyebrows, nasolabial folds, and central face, with or without pruritus [14].

Allergic Contact Dermatitis

Allergic contact dermatitis was considered when an eczematous dermatitis was associated with a positive patch-test reaction to a contact allergen and the reaction was considered clinically relevant based on sufficient exposure and compatibility of the dermatitis with the allergen in terms of clinical features, anatomical distribution, and the time course [15].

Irritant Contact Dermatitis

Irritant contact dermatitis was considered based on the presence of dermatitis with a compatible history of exposure to potential irritants, temporal relationship to the irritant, and distribution corresponding to the exposure site. Clinical features include burning or stinging, erythema, scaling, dryness, and fissuring [16].

Photoaggravated Dermatitis

This was considered when a pre-existing inflammatory dermatosis showed reproducible worsening following sunlight exposure. Lesions are seen predominantly photodistributed [17].

Airborne Contact Dermatitis (ABCD)

ABCD was considered when an acute or chronic eczematous eruption predominantly involved flexures or folds of exposed sites, particularly the face and neck, in the setting of a plausible exposure to airborne dust, pollens, sprays, fumes, or volatile chemicals [18]. Diagnosis was based on the correlation with exposure history, clinical morphology and distribution of lesions, and patch-test results.

Patients were also evaluated for alternative diagnoses, and potassium hydroxide examination and skin biopsy were performed in selected cases wherever indicated. Patients with active facial infections and concomitant active dermatoses were excluded. Although patient with connective tissue diseases (CTD) was not excluded as such, we did not come across any patient with CTD having facial dermatitis during the study period.

Data collection and clinical evaluation

A structured proforma was used to document the sociodemographic profile, duration of disease, sites involved, symptoms, aggravating factors, and history of atopy. Current and recent exposure to cosmetics, personal care products, hair products, jewellery, occupational agents, and other potential sources of facial exposure was documented at enrolment. Clinical diagnoses were established according to the criteria described above.

Medication washout periods

Medication washout periods were standardised before patch testing. Patients on immunosuppressants or oral prednisolone at doses > 20 mg/day were excluded. Oral corticosteroids were discontinued for three to four weeks before testing. In accordance with the 2026 European Society of Contact Dermatitis (ESCD) guideline [15], topical corticosteroids were discontinued at the patch test site for 7 days (for low to medium potency preparations) and 14 days (for high potency preparations). However, topical steroids could be continued on sites of active inflammation like the face, when clinically necessary. Antihistamines did not require a washout period.

Patch testing, interpretation, and clinical relevance assessment

Patch testing was performed using the Contact and Occupational Dermatoses Forum of India (CODFI)-approved Indian Standard Battery/Indian Standard Series along with the cosmetic and fragrance series [19]. A total of 50 allergens were tested using the CODFI-approved Indian Standard Battery together with the cosmetic and fragrance series. The allergens, their concentrations, and vehicles are detailed in Table 1.

**Table 1:** Patch-test allergens, concentrations, and vehicles

S. No.	Allergen	Concentration	Vehicle
1	Vaseline	100%	Petrolatum
2	Wool alcohol	30%	Petrolatum
3	Balsam of Peru		
4	Formaldehyde	2%	Petrolatum
5	Mercaptobenzothiazole	1%	Petrolatum
6	Colophony	20%	Petrolatum
7	Cobalt sulphate	2%	Petrolatum
8	Potassium dichromate	0.5%	Petrolatum
9	Nickel sulphate	5%	Petrolatum
10	Epoxy resins	1%	Petrolatum
11	Chlorocresol	1%	Petrolatum
12	Paraphenylenediamine (PPD)	1%	Petrolatum
13	Parthenium	15%	Petrolatum
14	Benzocaine	5%	Petrolatum
15	Neomycin sulfate	20%	Petrolatum
16	Paraben mix	9%	Petrolatum
17	Fragrance mix	8%	Petrolatum
18	Thiuram mix	1%	Petrolatum
19	Nitrofurozone	1%	Petrolatum
20	Black rubber mix	0.6%	Petrolatum
21	Vaseline	100%	Petrolatum
22	Ethylene diamine	1%	Aqueous
23	Benzyl alcohol	1%	Petrolatum
24	Benzyl salicylate	2%	Petrolatum
25	Bronopol	0.30%	Petrolatum
26	Butylated hydroxyanisole (BHA)	2%	Petrolatum
27	Butylated hydroxytoluene (BHT)	2%	Aqueous
28	Cetyl alcohol	5%	Petrolatum
29	Chloroacetamide	0.20%	Petrolatum
30	Geranium oil	2%	Petrolatum
31	2-Hydroxy-4-methoxybenzophenone	2%	Petrolatum
32	2-(2-Hydroxy-5-methylphenyl)benzotriazole	2%	Petrolatum
33	Imidazolidinylurea	2%	Petrolatum
34	Isopropyl myristate	20%	Petrolatum
35	Jasmine absolute	2%	Petrolatum
36	Lavender absolute	2%	Petrolatum
37	Musk mix	5%	Petrolatum
38	Phenyl salicylate	1%	Petrolatum
39	Polyoxyethylene oleate	2%	Petrolatum
40	Rose oil	2%	Petrolatum
41	Sorbitan sesquioleate	2%	Petrolatum
42	Thiomersal	0.1%	Petrolatum
43	Triclosan	2%	Petrolatum
44	Triethanolamine	2%	Petrolatum
45	Vanillin	2%	Petrolatum
46	Cetrimide	0.5%	Petrolatum
47	Hexamine	2%	Petrolatum
48	Chlorhexidine digluconate	0.5%	Petrolatum
49	Diazolidinylurea	2%	Aqueous
50	Propylene glycol	5%	Petrolatum

The patch-test materials were manufactured by Systopic Laboratories Private Limited, New Delhi, India, in collaboration with CODFl. Patients were advised to avoid topical medications, antihistamines, and corticosteroids during the testing period unless medically necessary. Nickel exposure was assessed by correlating the positive patch test reaction and clinical distribution of dermatitis with the patient’s documented exposure history, particularly jewellery, spectacle frames, and other metallic or cosmetic accessories. The patient's own cosmetic products like kajal, lipsticks, bindi, and sindoor were also tested, wherever required. 

We used Finn Chambers consisting of 10 aluminium wells per strip, with an inner diameter of 8 mm and a depth of 0.5 mm for each well. The selected allergens were applied into the respective chambers and affixed to the upper back under occlusion with standardised conditions. Patch test readings were taken at 48 and 96 hours, with the latter taken as the delayed reading. A day seven reading was not performed.

Reactions were graded according to the International Contact Dermatitis Research Group (ICDRG) criteria [20]. Positive reactions were then correlated with the exposure history, and their clinical relevance was assessed using the C=Current Relevance, O=Occupational, A=Accidental, D=Doubtful Relevance, E=Exposed, X=Unknown (COADEX) system, which classifies relevance as current, occupational, accidental, doubtful, exposed, or unknown [21]. A positive allergen was considered to have current relevance when a documented facial exposure pathway was identified, and the exposure was temporally compatible with the dermatitis. Reactions were classified as “possible” when the clinical and exposure information suggested, but did not establish relevance. Repeat open application testing (ROAT) was not routinely performed. Doubtful (+/−) reactions were not recorded as positive reactions, and no irritant reactions, excited skin syndrome, or active sensitisation reactions were observed during the study.

Psychosocial assessment

Psychosocial burden was assessed using the Skindex-16 questionnaire, a dermatology-specific quality-of-life instrument based on the established Skindex-16 framework developed for measuring the effect of skin disease on patients' symptoms, emotions, and functioning [9,10,12]. Total scores were categorised as mild (0-32), moderate (33-64), or severe (> 64). The cut-offs used in our study were arbitrary thresholds, and this categorisation was done to facilitate interpretation of the Skindex-16 scores and to explain the degree of psychosocial impact to patients in an easily understandable manner. Domain-wise scores for symptoms, emotional burden, and functioning were also recorded for all participants. 

A study by Nock et al. proposed severity strata for Skindex-16, providing further context for the interpretation of Skindex-16 scores. Nock et al. proposed domain-specific severity cut-offs for Skindex-16 in patients with acne [22]. Similarly, a Brazilian study by Cárcano et al. demonstrated differences in Skindex-16 scores across clinically defined mild, moderate, and severe disease groups; however, they did not establish specific numerical Skindex-16 cut-offs [23].

Statistical analysis

Statistical analysis was performed using Microsoft Excel (Microsoft Corporation, Redmond, Washington, United States) and IBM SPSS Statistics for Windows, Version 28.0 (IBM Corp., Armonk, New York, United States), which was used under an institutional license. Descriptive statistics were expressed as mean ± standard deviation for continuous variables and as frequencies and percentages for categorical variables. Associations between categorical variables were evaluated using Fisher's exact test. A two-tailed p-value of less than 0.05 was considered statistically significant. Additional exploratory analyses were performed to assess differences in Skindex-16 scores according to selected clinical and demographic variables. The Mann-Whitney U test was used for two-group comparisons, while the Kruskal-Wallis test with post-hoc Dwass-Steel-Critchlow-Fligner (DSCF) test was used for comparisons across more than two groups. Effect size was expressed using epsilon-squared (ε²) where appropriate.

Ethical considerations

Institutional Ethics Committee approval (IEC-3221) was obtained prior to study initiation. All participants were informed about the study objectives and procedures before enrolment, and written informed consent was obtained. Confidentiality of participant data was maintained throughout the study.

## Results

Clinical, sociodemographic profile and clinical presentation

Among the 50 enrolled patients, 37 (74%) were females, and 13 (26%) were males. The largest age group was 31-40 years, accounting for 20 patients (40%), followed by 41-50 years in 11 patients (22%). Most patients had chronic disease, with a duration of more than six months in 32 patients (64%). Housewives constituted the largest occupational subgroup, with 21 patients (42%). Fourteen patients (28%) reported a history of atopy. Cheek was the most frequent site of involvement, occurring in 42 (84%) patients, followed by the forehead in 38 (76%) patients and the eyelids in 34 (68%) patients. Pruritus was present in all 50 patients (100%), while redness and burning were also commonly seen in 40 (80%) and 36 (72%) patients, respectively. Dryness was seen in 18 (36%) participants. Hyperpigmentation was present in 17/50 (34%) participants. 

Aggravating factors and exposure profile

Sun exposure emerged as the predominant aggravating factor and was reported by 46 patients (92%), followed by heat or sweating in 28 (56%), seasonal variation in 27 (54%), hair dye exposure in 25 (50%), and dust exposure in 24 (48%). Hair oil, gel, or serum use was the most frequent overall exposure, seen in 47 (94%) participants, followed by jewellery use in 36 (72%), hair dye use in 31 (62%), household disinfectants in 26 (52%), and moisturisers or creams in 24 (48%).

Patch test findings

Positive patch test reactions were observed in 12/50 patients (24%), whereas 38/50 patients (76%) had negative results. The individual allergen profile has been listed in Table 2.

**Table 2:** Allergen-specific patch-test reactions, ICDRG grading, exposure sources, and COADEX relevance Percentages are calculated using the total number of allergen reactions (n = 15). 3 patients demonstrated sensitisation to two allergens each, resulting in 15 allergen reactions among the 12 patch-test-positive patients. COADEX: C = Cosmetic/Topical; E = Environmental/Occupational. Clinical relevance was classified as Present when supported by a documented facial exposure pathway and Possible when a plausible facial exposure route had indirect supporting evidence. COADEX: C=Current Relevance, O=Occupational, A=Accidental, D=Doubtful Relevance, E=Exposed, X=Unknown; ICDRG: International Contact Dermatitis Research Group.

Allergen	No. of reactions (n)	Percentage (%)	ICDRG grade	Suspected exposure source	COADEX	Clinical relevance
Paraphenylenediamine (PPD)	4	26.7	Grade 1+ (4)	Hair dyes, hair colourants, salon hair products	C	Present
Benzyl alcohol	2	13.3	Grade 1+ (1), Grade 2+ (1)	Cosmetics, creams, topical preparations	C	Present
Benzyl salicylate	2	13.3	Grade 1+ (1), Grade 2+ (1)	Perfumes, sunscreens, facial creams, cosmetics	C	Present
Bronopol	2	13.3	Grade 1+ (1), Grade 2+ (1)	Moisturisers, face washes, homemade/Ayurvedic creams	C	Present
Nickel sulphate	1	6.7	Grade 2+ (1)	Jewellery, spectacle frames, cosmetic accessories, bindi clips	C	Present
Wool alcohol (Lanolin)	1	6.7	Grade 1+ (1)	Moisturisers, creams, ointment bases	C	Possible
Sindoor/topical cream	1	6.7	Grade 1+ (1)	Sindoor, coloured topical facial cosmetics	C	Present
Lipstick	1	6.7	Grade 1+ (1)	Lipstick, tinted lip products, decorative cosmetics	C	Present
Parthenium hysterophorus (Parthenolide)	1	6.7	Grade 1+ (1)	Airborne pollen, weed/outdoor exposure	E	Present
Total	15	100.0	—	—	—	—

Grade 1+ reactions were seen in 10 patients (20%), and grade 2+ reactions were seen in two patients (4%). No grade 3+ reactions were identified. Among the age groups, patch test positivity was highest among the 31-40 years and 41-50 years groups, with four patients (8%) in each group showing positive patch test results. However, no statistically significant association was found between patch-test positivity and age group (p = 0.753), gender (p = 1.000), marital status (p = 0.379), or occupation (p = 0.081) on Fisher’s exact test. 

Hyperpigmentation was present in 17/50 (34%) participants. It was observed in all 12/12 (100%) patch-test-positive patients compared with 5/38 (13.2%) patch-test-negative patients, with a statistically significant association (Fisher’s exact test, p < 0.001). Patch-test positivity was also significantly associated with hair-dye use, with all 12 patch-test-positive patients reporting hair dye usage compared with 19/38 (50.0%) patch-test-negative patients (Fisher’s exact test, p = 0.002). A significant association was also observed between patch-test positivity and history of atopy, which was present in 7/12 (58.3%) patch-test-positive patients compared with 7/38 (18.4%) patch-test-negative patients (Fisher’s exact test, p = 0.023). There was no significant association between patch-test positivity and duration of facial dermatitis (Fisher’s exact test, p = 0.191). Dryness was seen in 3/12 (25%) patch-test-positive cases.

The Skindex-16 total score was not normally distributed (Shapiro-Wilk W = 0.774, p < 0.001), therefore, non-parametric tests were used for group comparisons. Skindex-16 total scores did not differ significantly by gender (median 35.0 in females vs. 34.0 in males; Mann-Whitney U = 188, p = 0.238). In contrast, patch-test-positive patients had significantly higher Skindex-16 total scores than patch-test-negative patients (median 69.0 vs. 34.0; Mann-Whitney U = 2.00, p < 0.001; rank-biserial correlation = 0.991). Patients with hyperpigmentation also had significantly higher Skindex-16 total scores than those without hyperpigmentation (60.7 ± 14.7 vs. 28.4 ± 8.0; Mann-Whitney U = 16.0, p < 0.001; rank-biserial correlation = 0.943). Finally, Skindex-16 total scores differed significantly across disease duration groups (Kruskal-Wallis χ² = 17.3, df = 2, p < 0.001; ε² = 0.353). Post hoc DSCF comparisons demonstrated significant differences between the 1-6 month and > 6 month groups (p < 0.001) and between the < 1 month and > 6 month groups (p = 0.042), but not between the < 1 month and 1-6 month groups (p = 0.393). Given that the < 1-month group comprised only three patients, these findings have been interpreted cautiously.

Allergen profile and clinical relevance

A total of 15 allergen reactions were identified in 12 patch-test-positive patients, and three patients demonstrated co-sensitisation to two allergens each. Paraphenylenediamine was the most common allergen seen in 4/15 (26.7%) positive allergen reactions, followed by benzyl alcohol, benzyl salicylate, and bronopol, each having 2/15 (13.3%) positive allergen reactions. Wool alcohol, Nickel sulphate, Parthenium, lipstick, and sindoor were each seen in one (6.7%) patch test-positive participant. No positive reaction to fragrance mix was observed. According to the COADEX system, of the nine distinct allergen types identified, eight (approximately 89%) were classified as Cosmetic/Topical (COADEX C), while one was classified as Environmental (COADEX E).

Psychosocial burden

Based on the Skindex-16 total score, moderate psychosocial impairment was the most common category and was observed in 25 (50%) participants. Severe psychosocial impact was noted in 13 (26%) participants, while 12 (24%) participants showed mild psychosocial impairment (Table 3). Patients with hyperpigmentation had significantly higher total Skindex-16 scores than those without hyperpigmentation (60.7 ± 14.7 vs. 28.4 ± 8.0, respectively; Mann-Whitney U = 16.0, p < 0.001). Similarly, the emotional-domain scores were significantly higher among patients with hyperpigmentation [median (IQR): 32.4 (0.5) vs. 16.2 (7.6); Mann-Whitney U = 16.0, p < 0.001]. Emotional domain scores were slightly higher among females than males [median (IQR): 16.7 (16.2) vs. 16.2 (0.0), respectively]; however, the difference was not statistically significant (Mann-Whitney U = 188, p = 0.238). Total Skindex-16 scores also differed significantly across disease-duration groups (Kruskal-Wallis H = 17.3, df = 2, p < 0.001; ε² = 0.353). Post-hoc DSCF analysis demonstrated significant differences between the 1-6-month and > 6-month groups (p < 0.001) and between the < 1-month and > 6-month groups (p = 0.042). Given the small number of patients in the < 1-month group (n = 3), these findings were interpreted cautiously.

**Table 3:** Distribution of Skindex-16 total score categories among study participants

Skindex-16 total score category	Score range	Number n (%)
Mild psychosocial impact	0 to 32	12 (24%)
Moderate psychosocial impact	33 to 64	25 (50%)
Severe psychosocial impact	More than 64	13 (26%)

The emotional domain had the highest mean score, at 18.8 ± 8.9. The functional domain showed a mean score of 11.2 ± 5.3, while the symptoms domain had a mean score of 9.4 ± 4.5. The mean total Skindex-16 score was 39.4 ± 18.7 (Table 4).

**Table 4:** Domain-wise mean Skindex-16 scores and percentage of maximum possible score among study participants Each Skindex-16 item is scored from 0 to 6. The percentage of maximum possible score was calculated descriptively as (mean score / maximum possible domain score) × 100. (Note: These percentages are not separate validated Skindex-16 scoring thresholds).

Skindex-16 domain	Number of items	Maximum possible score	Mean score ± SD	Mean score (% of maximum possible score)
Symptoms	4	24	9.4 ± 4.5	39.2%
Emotional	7	42	18.8 ± 8.9	44.7%
Functional	5	30	11.2 ± 5.3	37.3%
Total Skindex-16	16	96	39.4 ± 18.7	41.0%

## Discussion

Facial dermatitis is clinically important because it represents a visible and often chronic dermatosis with multifactorial triggers, including cosmetic, irritant, allergic, airborne, and environmental factors. Even limited facial involvement may produce disproportionate psychosocial distress because of the cosmetic and social relevance of the face [2-6,11-13]. 

The clinical pattern was characterised by frequent involvement of the cheeks, forehead, and eyelids. These sites are especially susceptible because they are repeatedly exposed to direct and indirect contactants, including facial preparations, hair products, and airborne allergens [3-6,9,10]. 

The present study demonstrated that facial dermatitis predominantly affected females, with housewives representing a majority of the study population. This may be due to the fact that women in socially active adult age groups often show greater disease burden because of more frequent exposure to cosmetics, hair dyes, fragrances, moisturisers, and topical facial products [5,6,9,10] and thus have been over-represented in our study. Among age groups, positive tests were most commonly seen in the 31-40 years age group. There was no statistically significant association observed between patch-test positivity and gender, marital status, or occupation. Although positive reactions were numerically more frequent among females, married participants, and housewives, these differences were not statistically significant on Fisher’s exact test (p = 1.000, 0.379, and 0.081, respectively). 

In darker skin phototypes, recurrent inflammation and contact dermatitis may lead to persistent post-inflammatory hyperpigmentation. Previous Indian work has specifically evaluated patch-test positivity in patients with facial pigmented contact dermatitis [24]. In the present study, hyperpigmentation was observed in 34% of participants and was significantly associated with patch-test positivity. Patients with hyperpigmentation also had higher total Skindex-16 scores, indicating greater psychosocial burden, which may reflect the significant role of facial appearance in self-image and social interaction.

Patch test positivity was observed in 24% of patients. Paraphenylenediamine was the most common allergen. This finding is clinically plausible because hair dye use was common in our study, and transfer of hair dye to the forehead, hairline, ears, eyelids, and neck can lead to allergic contact dermatitis at these sites [5,6,10]. Other identified allergens, including benzyl alcohol, benzyl salicylate, bronopol, wool alcohol, and nickel sulphate, were associated with exposures to preservatives, fragrance components, emollient bases, and metallic products [5-10].

The present study had comparatively lower patch test positivity rates compared to several other studies on facial or cosmetic contact dermatitis. Yadav et al. reported 65.5% positivity among patients with facial contact dermatitis, while Rastogi et al. reported a positivity rate of 68% among patients with suspected cosmetic-induced facial dermatoses [6, 9]. Kumar et al. reported 76% confirmed cosmetic allergic contact dermatitis among patients suspected of having cosmetic allergy [25]. Internationally, Kasemsarn et al. reported at least one positive reaction in 81.6% of facial dermatitis patients referred to a contact dermatitis clinic [3]. The relatively lower positivity in our cohort may partly reflect the heterogeneous etiology of facial dermatitis in our study, as the study was not restricted to patients with a high clinical suspicion of allergic contact dermatitis. Irritant contact dermatitis and other non-allergic inflammatory dermatoses may therefore have contributed to patch test-negative cases. In our cohort, sun exposure (92%), heat/sweating (56%), seasonal variation (54%), and dust exposure (48%) were common aggravating factors, suggesting a possible role of non-allergic environmental triggers in a proportion of patients. 

The COADEX system-based relevance assessment showed that cosmetic or topical sources accounted for 89% of the identified allergens. This finding highlights the importance of taking a detailed exposure history in patients with facial dermatitis, asking the patient about usage of moisturisers, creams, homemade or Ayurvedic preparations, hair dyes, cosmetics, and household products [4-10]. Such evaluation helps in counselling the patients on allergen avoidance and giving them suitable alternatives [4-10]. 

The present study demonstrated appreciable psychosocial burden among patients with facial dermatitis, with a mean total Skindex-16 score of 39.4 ± 18.7. The emotional domain had the highest mean score (18.8 ± 8.9), followed by functioning (11.2 ± 5.3) and symptoms (9.4 ± 4.5). This predominance of emotional burden is consistent with findings in acne, melasma, and visible eczema, where facial visibility and its effects on self-image and social interactions contribute substantially to quality-of-life impairment [26-28]. Facial erythema, scaling, and pigmentary changes may influence self-confidence and raise concerns about social perception. Recurrent flares, uncertainty regarding triggers, and the need to modify cosmetic and personal-care practices may further contribute to emotional distress. This may explain why psychosocial burden can be considerable even when the anatomical extent of disease is limited. In our study, patients with hyperpigmentation also had significantly higher total and emotional-domain Skindex-16 scores, further highlighting the psychosocial relevance of visible pigmentary changes. Emotional-domain scores did not differ significantly by gender. These findings highlight the importance of incorporating psychosocial assessment in patients with facial dermatitis.

A notable finding was that 76% of participants had negative patch-test results. A negative patch test does not exclude facial dermatitis and does not necessarily indicate the absence of an external trigger. First, some patients may have had irritant contact dermatitis. Second, the heterogeneous population may have included endogenous causes like atopic dermatitis or seborrheic dermatitis, in which patch testing would not necessarily identify a causative allergen. Third, photoaggravated dermatitis is also a possibility, because sunlight was a frequent aggravating factor in our cohort. Other explanations include airborne contact dermatitis, unidentified environmental or occupational exposures, and sensitisation to allergens not represented in the Indian Standard Battery or Cosmetic and Fragrance Series. When evaluating a patient with facial dermatitis, a thorough clinical assessment, detailed exposure history, patch testing, identification of relevant allergens, counselling on allergen avoidance, and consideration of psychosocial morbidity are important [4-6,11-17].

Limitations

The relatively small sample size may have limited the statistical power to detect significant associations. As this was a single-centre study conducted at a tertiary care institution, the findings may not be fully generalisable to the broader population. Photopatch testing was not performed, so the contribution of photoallergic contact dermatitis could not be evaluated. In addition, allergens not included in the standard patch test series, particularly less common cosmetic ingredients, may have remained undetected. Finally, inclusion of only regular users of cosmetic or personal-care products may have skewed exposure patterns and reduced the generalisability of our findings, as there are patients with facial dermatitis who do not regularly use such products. The mild, moderate, and severe Skindex-16 categories used in this study were study-defined thresholds intended to facilitate descriptive interpretation and have not been validated for facial dermatitis. 

## Conclusions

In our cohort, facial dermatitis was most frequently observed among women in the middle-aged group. Patch testing identified contact sensitisation in 24% of patients and helped identify clinically relevant allergens, predominantly related to cosmetic and personal care products. Environmental aggravating factors such as sun exposure, heat, and dust were also frequently reported, which may contribute to worsening of facial dermatitis. Our study also demonstrated a measurable psychosocial burden using Skindex-16 scores, especially in the emotional domain. Hyperpigmentation was significantly associated with greater psychosocial burden. Patch testing can assist in identifying clinically relevant contact sensitisation and may help guide allergen avoidance; however, longitudinal studies are needed to determine whether patch testing and subsequent allergen avoidance improve long-term clinical outcomes.
